# QbD Approach for Development of a Mucoadhesive Thermosensitive Gel for Oral Application: Risk Assessment Followed by Screening and Optimization

**DOI:** 10.3390/gels12040331

**Published:** 2026-04-16

**Authors:** Elena Dinte, Ioan Tomuță, Rareș Iuliu Iovanov, Tibor Casian, Ana Marcela Achim, Aranka Ilea, Adina Bianca Bosca, Horațiu Rotar

**Affiliations:** 1 Department of Pharmaceutical Technology and Biopharmacy, Faculty of Pharmacy, “Iuliu Haţieganu” University of Medicine and Pharmacy, 41 V. Babes Street, 400012 Cluj-Napoca, Romania; edinte@umfcluj.ro (E.D.); riovanov@umfcluj.ro (R.I.I.); casian.tibor@umfcluj.ro (T.C.); machim@umfcluj.ro (A.M.A.); 2 Department of Oral Rehabilitation, Faculty of Dentistry, “Iuliu Hațieganu” University of Medicine and Pharmacy, 400012 Cluj-Napoca, Romania; aranka.ilea@umfcluj.ro; 3 Department of Morphological Sciences, Faculty of Medicine, “Iuliu Hațieganu” University of Medicine and Pharmacy, 400012 Cluj-Napoca, Romania; biancabosca@umfcluj.ro; 4 Department of Oral and Cranio-Maxillofacial Surgery, Faculty of Dentistry,” Iuliu Hațieganu” University of Medicine and Pharmacy, 400012 Cluj-Napoca, Romania; horatiu.rotar@umfcluj.ro

**Keywords:** alveolar osteitis, mucoadhesion, thermosensitive hydrogel, optimization, Quality by Design

## Abstract

The study aimed to develop a mucoadhesive thermosensitive buccal gel capable of forming an artificial clot after application in the extraction socket and providing prolonged release for metronidazole (MZ) and ibuprofen (IB). The critical quality attributes of the product were systematically evaluated using Ishikawa (cause–effect) diagrams as a risk assessment tool, considering the factors related to the formulation, process, and methodology. Subsequently, Failure Mode and Effects Analysis (FMEA) was used to identify the critical parameters of the formulation and process characterized by a high probability of occurrence and a significant impact on product performance. The influence of qualitative and quantitative formulation variables was further investigated using two experimental designs, applied for both screening and optimization purposes. The rheological, adhesion, and in vitro release properties of the drugs were studied, and the optimized formulation for these characteristics contains Poloxamer 407 20.99% and HPMC K100M:K4M 1:1, 0.74%. The release of MZ and IB was prolonged over 8 h and followed Peppas’s kinetics. The optimized formula had an appropriate pH and an acceptable ex vivo mucoadhesion time. Stability studies revealed the preservation of mechanical properties and a recovery coefficient for MZ and IB of over 90%, after 12 months of storage. The optimized formula may be a potential candidate for the prevention of alveolar osteitis.

## 1. Introduction

Oral health statistics highlight a global public health problem, with nearly 3.7 billion people affected by oral diseases, with a negative impact on quality of life [1]. In addition to dental caries and periodontal disease, some oral lesions are transient and of local cause (local trauma), whereas others are secondary to local or systemic conditions (oral cancer, oral lichen planus, stomatitis, gingivitis of various etiologies), including iatrogenic implications [2,3].

Alveolar osteitis (AO), commonly called “dry socket”, is a debilitating, painful condition and one of the most common complications after tooth extraction. It begins after approximately 3–7 days and has an incidence that varies between 0.5% and 4% but can reach up to 30% in the case of extractions of mandibular third molars [4,5,6]. AO occurs because of the absence of a blood clot after extraction or by premature fibrinolytic decomposition of the formed clot. This exposes the underlying bone in the extraction socket to the septic oral environment, favoring inflammation [7]. Etiology is multifactorial, represented by local and systemic factors, and an important role can also be attributed to the local microbiota, the most common bacteria identified in patients with alveolar osteitis being Fusobacterium, Parvimonas, Prevotella, and Peptostreptococcus [8]. Risk factors for the development of AO are represented by poor oral hygiene, smoking, age, administration of certain medications (vasoconstrictors, bisphosphonates, corticosteroids, oral contraceptives), but also some chronic diseases, such as type 2 diabetes mellitus. The symptomatology is nonspecific, represented by prolonged pain and halitosis, associated with signs of inflammation [5,9].

AO treatment aims to diminish inflammation, reduce pain, heal lesions, and consists of the local application of antiseptics, antibacterials, anti-inflammatories, analgesics, or the use of physiotherapeutic methods, without establishing standardized protocols. Although AO is not considered an infectious condition and oral treatment with pre- or post-extraction antibiotics is not recommended, local treatment can have an important role in prevention and should be instituted in patients at risk [6]. Thus, oral and maxillofacial surgeons can reduce the severity and incidence of AO through personalized, patient-centered approaches; in general, dressings based on eugenol, thymol, zinc oxide, chlorhexidine, etc., can be applied to the extraction socket, being available in the form of gels, fibers (Alveogyl^®^), cones (Neocone^®^) or pastes (Socketol^®^), depending on the patient’s sensitivity to the active components, but the existing products do not cover the needs of medical practice [7,10,11,12].

The use of a semi-solid, hydrogel-type preparation in the extraction socket presents multiple advantages, such as easy application and maintenance of APIs directly on the affected area; no need to remove the preparation, as it is gradually eluted with saliva; and the possibility of incorporating a high concentration of APIs, so that therapeutic efficiency is ensured [11]. The critical quality characteristics for a preparation applicable in the oral cavity are represented by the adhesiveness of the preparation to the mucosa, which determines an increased residence time at this level, ensuring a prolonged release of the incorporated APIs and, consequently, a prolonged therapeutic effect. Thus, the product design will consider the selection of bioadhesive materials that offer increased consistency and viscosity, ensuring resistance to shear forces generated by physiological activities in the oral cavity. In addition, the preparation must have an adequate spreading capacity to be easily applied in different areas of the oral cavity that are more difficult to approach, such as application in the extraction socket. Also, the preparation must demonstrate good tolerance to the oral mucosa: adequate pH, homogeneity, and a lack of irritating action of the components; in addition, the preparation must ensure the physicochemical stability of the incorporated APIs during storage and maintain its mechanical characteristics, which may affect its viscosity and bioadhesive capacity.

Formulations of buccal mucoadhesive systems containing bioadhesive polymers (polyacrylic acid derivatives, polyethylene oxides, cellulose derivatives) have been reported. They are presented in the form of films [13], oleogels [14], or hydrogels, with increased interest in thermosensitive hydrogels [15,16]. Poloxamer 407 is a nonionic hydrophilic triblock copolymer, formed by polypropylene glycol and polyethylene glycol units, whose aqueous solution undergoes a temperature-dependent liquid–gel phase transition. In combination with bioadhesive polymers, it can form stable, biodegradable gels that provide prolonged API release. Hydroxypropylmethylcellulose (HPMC) is a well-known excipient with multiple uses in the preparation of controlled-release and mucoadhesive dosage forms [17]. Metronidazole is an antimicrobial active against anaerobic bacteria that inhabit the oral cavity [18], and ibuprofen has a significant role in reducing chronic pain and inflammation [19].

Despite the growing interest in localized therapies for OA, the development of topical formulations in this field has rarely been conducted within a structured Quality by Design (QbD) framework. Most currently available dressings and semi-solid preparations are formulated empirically, without systematic risk assessment, multivariate optimization, or a regulatory-oriented definition of critical quality attributes. Furthermore, although mucoadhesive hydrogels have been widely investigated for buccal drug delivery, the establishment of a statistically validated design space for thermosensitive mucoadhesive gel remains limited in the literature, particularly with respect to formulations intended for post-extraction socket applications. In addition, while metronidazole and ibuprofen are individually used in oral inflammatory conditions, their combined incorporation into a single thermoreversible, in situ-gelling mucoadhesive system designed to provide antimicrobial and anti-inflammatory effects simultaneously has not been comprehensively explored. Therefore, a rationally designed thermosensitive gel developed under a QbD paradigm, integrating risk assessment, multivariate experimental design, and design space definition, may represent a meaningful advancement in the management of alveolar osteitis.

This study aimed to develop and characterize a novel mucoadhesive thermosensitive buccal gel formulation (MZ-IB-MTBG) capable of forming a stable artificial clot after application to the extraction socket and ensuring a prolonged release of MZ and IB.

## 2. Results and Discussion

The development of a thermosensitive buccal mucoadhesive gel is a multifactorial process that requires the simultaneous optimization of formulation composition (active pharmaceutical ingredient, polymer concentration, polymer ratios, plasticizers/penetration enhancers) and material performance (component compatibility, rheological and bioadhesive behavior, or mucoadhesive strength). These elements must be carefully balanced to ensure consistent manufacturing, predictable in situ gel formation, and reproducible therapeutic performance. In this context, the thermosensitive gel formulation was designed through a systematic evaluation of formulation and process variables that may influence the critical quality attributes of the final product (spreadability, viscosity at 25 °C and 37 °C, bioadhesion, and in vitro release). Those parameters need to be assessed to ensure that the final formulation meets the quality attributes needed for its intended use. A systematic evaluation can be conducted using a Quality by Design (QbD) approach.

### 2.1. The QbD Approach in the Development of a Mucoadhesive Thermosensitive Buccal Gel Formulation

#### 2.1.1. Establishment of QTTP and CQAs

The first step in applying the Quality by Design (QbD) framework to the development of a thermosensitive buccal mucoadhesive gel is to clearly define the desired Quality Target Product Profile (QTPP). Subsequently, the critical quality attributes (CQAs)—representing those physicochemical, rheological, and performance characteristics with the greatest impact on product safety, efficacy, and clinical performance—must be systematically identified [20].

The QTPP and the derived CQAs were established based on scientific rationale, regulatory expectations, and practical formulation constraints specific to semi-solid preparation. Particular consideration was given to attributes such as spreadability, viscosity profile, mucoadhesive strength, drug content, pH compatibility with the buccal mucosa, and in vitro drug release behavior [14].

A summary of the targeted QTPP is presented in Table 1, designed with the objective of developing a drug-loaded thermosensitive buccal gel capable of undergoing rapid gelation at buccal temperatures (≈32–37 °C), exhibiting prolonged mucosal residence time, and ensuring a controlled and reproducible drug release profile under simulated buccal conditions.

#### 2.1.2. Risk Identification and Evaluation

The critical quality attributes (CQAs) derived from the Quality Target Product Profile (QTPP) were primarily associated with product application and retention in the buccal cavity, specifically spreadability, viscosity, and mucoadhesive strength. These attributes are essential to ensure adequate administration, prolonged residence time, and consistent therapeutic performance [14,20].

**Figure 1:**
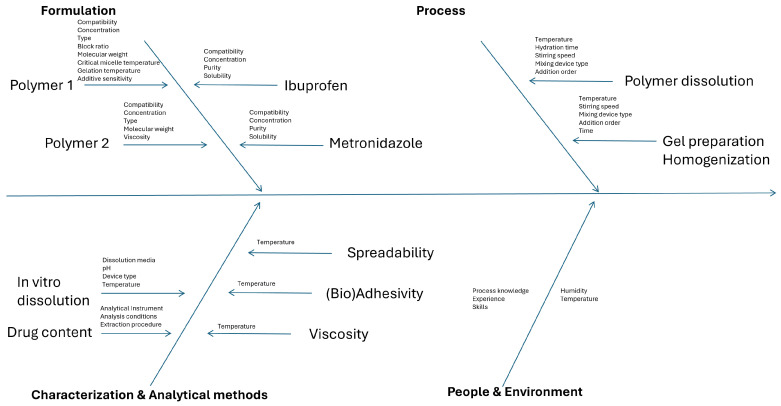
Ishikawa diagram summarizing the factors that could impact the quality of mucoadhesive thermosensitive buccal gel formulation.

Figure 1 presents the Ishikawa diagram summarizing formulation-, process-, characterization-, and methodology-related factors that may influence the selected CQAs.

Formulation variables were categorized into polymer- and active pharmaceutical ingredient (API)-related factors. For polymers, key physicochemical properties, including polymer type, molecular weight, gelation temperature, and concentration, were identified as critical determinants of the gel’s rheological behavior, adhesive performance, and uniformity. Polymer selection and optimization were associated with elevated RPN, underscoring their significant impact on product quality.

The literature data demonstrates that the mucoadhesive performance, gel strength, gelation temperature, viscosity, and drug release profiles of thermosensitive gels are strongly influenced by the choice and ratio of thermoresponsive and bioadhesive polymers. In this context, the selection of polymer type was further investigated by comparing polyethylene oxide (PEO 1105) and hydroxypropyl methylcellulose (HPMC K100M) as bioadhesive components within thermosensitive P407-based gel systems.

Poloxamer 407/poly(ethylene oxide)–poly(propylene oxide)–poly(ethylene oxide) triblock copolymers form temperature-responsive gels via micellization and gelation, with the gelation temperature being concentration- and composition-dependent. The incorporation of polymeric additives, including PEO, can alter gel properties with an effect on gelation thresholds and rheology, and gel integrity. PEO can modulate gelation by bridging micelles or changing water structure around EO segments [21,22,23]. HPMC K100M is a high-viscosity, high-molecular-weight cellulose ether used to impart mucoadhesion, viscosity enhancement, and controlled release in combination with thermoresponsive polymers. Higher viscosity grades of HPMC yield stronger gel networks and greater mucoadhesion via hydrogen bonding and chain entanglement with mucin glycoproteins [24]. The addition of HPMC can lower transition (sol–gel) temperatures towards physiological values while simultaneously enhancing viscosity and gel strength through interactions and polymer chain entanglement with the PEO-rich micellar network [23,25]. Regarding the drug release mechanism, PEO/Pluronic-dominant systems are diffusion-dominated, having also polymer dissolution/erosion (achieved by viscosity and gel strength) as a contributing mechanism. HPMC addition can further hinder diffusion and promote extended release [26,27,28].

A Design of Experiments (DoE) approach was employed to systematically evaluate the influence of PEO and HPMC on formulation performance, while maintaining Poloxamer P407 as the thermosensitive matrix-forming agent. Differences in polymer chain flexibility, hydration behavior, and viscosity contribution were expected to impact mucoadhesion and gel strength. These distinctions highlight the critical role of polymer type in balancing mucoadhesive performance and rheological properties, thereby reinforcing its high-risk ranking in the formulation development process.

API-related variables were mainly linked with solubility and concentration. Complete solubilization of the API within the gel matrix is essential to ensure homogeneity, reproducible drug release, and consistent therapeutic efficacy. These risks can be effectively mitigated by determining API solubility limits, avoiding drug loading above saturation levels, and selecting excipients that enhance solubilization.

Process parameters were grouped according to the preparation stages, namely polymer dissolution and homogenization. Experimental conditions, such as temperature, hydration time, order of component addition, homogenization speed, and mixing duration, were identified as potential sources of variability affecting final product performance. Implementation of optimized process conditions and standardized operating procedures can significantly reduce the associated risks and improve batch-to-batch consistency. Characterization and methodology-related factors require testing conditions that are relevant and representative of in vivo conditions (buccal environment), as this will guide product optimization in a direction that is aligned with clinical performance expectations.

For each identified parameter, the RPN was determined based on prior knowledge and experimental experience.

To ensure a consistent risk evaluation process, the Failure Mode and Effects Analysis (FMEA) scoring was performed using predefined qualitative criteria for occurrence (O), severity (S), and detectability (D), in accordance with ICH Q9 [29]. Each criterion was scored from 1 to 5, where higher values indicate greater risk.

Occurrence reflects the likelihood of a failure mode, severity represents the potential impact of the failure on CQAs, while detectability describes the ability to identify the failure mode prior to product use through in-process controls or post-formulation characterization. Polymer-related parameters (polymer type, concentration, molecular weight, and gelation temperature) were assigned with similar occurrence (O = 5) and severity (S = 5) scores due to their well-established critical influence on formulation performance. For these factors a moderate level of detectability was assigned (D = 3), as deviations in these attributes cannot be directly observed during processing but can be reliably identified through characterization methods such as rheological analysis, gelation temperature determination, and mucoadhesion testing. Consequently, identical RPN values (75) were obtained, reflecting their comparable criticality.

In contrast, API-related factors were assigned with lower occurrence and severity scores, as these risks can be effectively mitigated through preliminary solubility studies and appropriate formulation design. Process parameters were associated with lower RPNs, since deviations can be identified and controlled through standardized operating procedures and in-process evaluation. Similarly, characterization-related factors were assigned with lower occurrence scores, justified by the availability of well-established testing methodologies reported in the literature for thermosensitive and mucoadhesive gel systems, resulting in overall lower RPN values.

Variables with RPN values greater than 40 were considered high-risk and were subsequently selected as independent factors in the Design of Experiments (DoE) study (Table 2).

### 2.2. Design of Experiments (DoEs)

#### 2.2.1. Screening DoE

The first objective was to select a bioadhesive polymer and its concentration to be associated with P407 to prepare a vehicle with thermosensitive mucoadhesive properties, in which APIs with anti-inflammatory and antimicrobial action would be incorporated. Thus, the first experimental plan for the screening propose was designed; accordingly, gel base formulations were prepared, consisting of a thermosensitive polymer, P407, which was associated with polymers with known bioadhesive properties, PEO 1105 and HPMC K100M. The results obtained after preparing and analyzing the formulations are presented in Table 3. The data were fitted using Projection to Latent Structures by Partial Least Squares (PLS), and the performance was calculated. All PLS models demonstrated adequate model validity (validity > 0.25), indicating that none of the evaluated responses showed evidence of lack-of-fit. The high reproducibility values (>0.9) confirm that the experimental design was appropriate and that comparable outcomes were consistently obtained across replicate runs under identical conditions. Furthermore, the models adequately explained the variability of the responses (R^2^ > 0.8) and exhibited strong predictive performance (Q^2^ > 0.5), supporting their suitability for interpreting and predicting future observations [30].

##### The Influence of the Formulation Factors on Rheological and In Vitro Adhesive Characteristics of Gel Bases Formulations, Consisting of P407 Combined with PEO1105 and HPMC K100M

**Figure 2:**
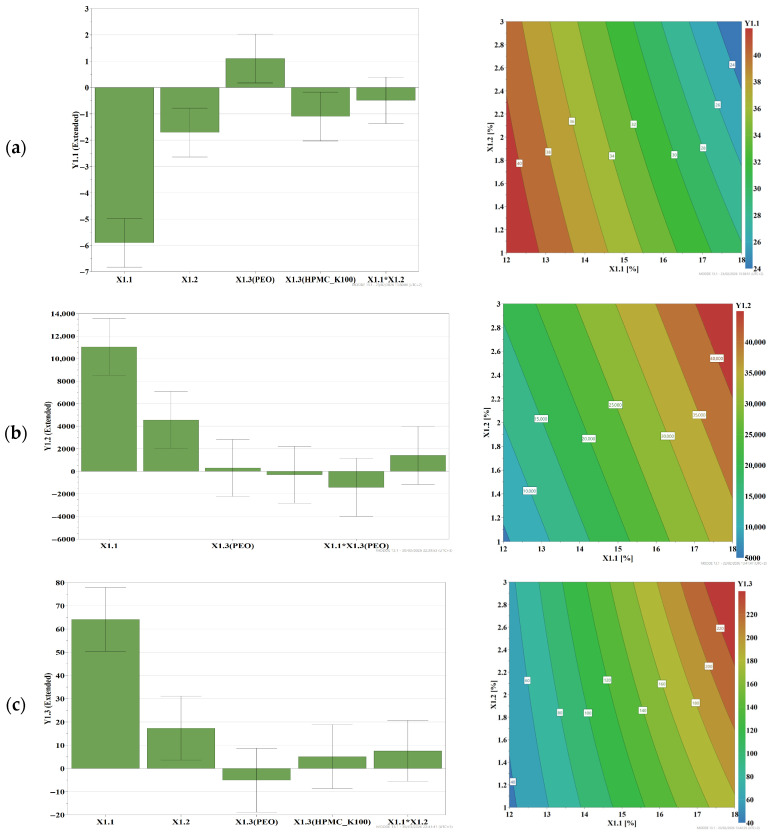
The influence of input variables on (**a**), spreadability—Y1.1; (**b**), viscosity at 37 °C—Y1.2; and (**c**), detachment force—Y1.3, according to screening DoE.

The experimental data on spreading, viscosity, and adhesion in vitro were fitted to a second-degree polynomial model, and the quantitative effects of the formulation factors on the experimental responses were illustrated by histograms and response surfaces (Figure 2).

Regarding the influence of formulation factors on the responses studied, the results showed that spreadability varied between 20.5 ± 1.8 mm and 42 ± 2.98 mm and decreased with increasing concentrations of P407 and bioadhesive polymers, HPMC K100M and PEO 1105, and this result is explained by the effect of polymers on increasing consistency, which reduces spreadability. In addition, PEO 1105 has a spreadability-increasing effect, by destructuring the P407 matrix and reducing consistency, while HPMC K100M leads to more consistent gels in mixture with P407, which reduces spreading. There were no interaction phenomena (Figure 2a).

The viscosity data studied at 37 °C showed an increase in this parameter with increasing polymer concentrations in the formulation, with a pronounced effect in the case of P407, which forms a gel with increasing temperature, with values between 2943 ± 456 mPa·s and 52,184 ± 1823 mPa·s. Although increasing the concentration of HPMC K100M or PEO 1105 causes an increase in viscosity, the type of polymer has no significant influence. There were no interaction phenomena (Figure 2b).

The in vitro detachment force was positively influenced by increasing the concentration of polymers used in the preparation, which had an increasing effect on the response, with values between 26 ± 2.1 mN and 250 ± 35 mN, and no interaction effects were observed (Figure 2c). In this case, P407 also showed a predominant effect.

#### 2.2.2. Optimization DoE

The second objective was to formulate a mucoadhesive buccal gel, loaded with MZ and IB, that would form an artificial clot after application to the extraction socket. Based on the results obtained from the screening carried out in the first experimental plan, the formulation based on P407 21% and HPMC K100M 0.5% showed suitable viscosity and adhesion properties to serve as a vehicle for APIs. Thus, P407 can rapidly form a stable gel after application to the extraction socket, and its residence in the oral cavity will be influenced by the bioadhesive polymer used, which confers mechanical resistance by increasing consistency and viscosity. As a result, the need to increase the concentration of the bioadhesive polymer was highlighted. Since HPMC K100M has high viscosity, to facilitate obtaining homogeneous formulations, it was chosen to incorporate mixtures of HPMC K4M:HPMC K100M, mixed in a 1:1 ratio; the total concentration of the mixture that can be used is 0.8%.

As a result, in order to optimize the formulation, a full factorial experimental design was constructed, which consisted of 12 runs (nine different formulations and one triplicated center point), in which P407 was combined in different concentrations (in the range of 18–21%) with different concentrations of hydroxypropyl methylcellulose (in the range of 0.2–0.8%), in the form of HPMC K4M:HPMC K100M mixtures, in a ratio of 1:1 (Table 9). The independent variables of the experimental design were represented by the concentrations of polymers used in the formulation (Table 9), and the dependent variables were the rheological, in vitro adhesion, and in vitro release studied characteristics.

Similar to screening, the experimental data (Table 4) were fitted using Projection to Latent Structures by Partial Least Squares (PLS), and the performance was calculated. All PLS models demonstrated adequate model validity (validity > 0.3), indicating that none of the evaluated responses showed evidence of lack-of-fit. The high reproducibility values (>0.7) confirm that the experimental design was appropriate and that comparable outcomes were consistently obtained across replicate runs under identical conditions. The resulting models captured most of the response variability (R^2^ > 0.8) and exhibited strong predictive performance (Q^2^ > 0.5), supporting their suitability for interpretation and their reliability for process understanding and optimization.

**Figure 3:**
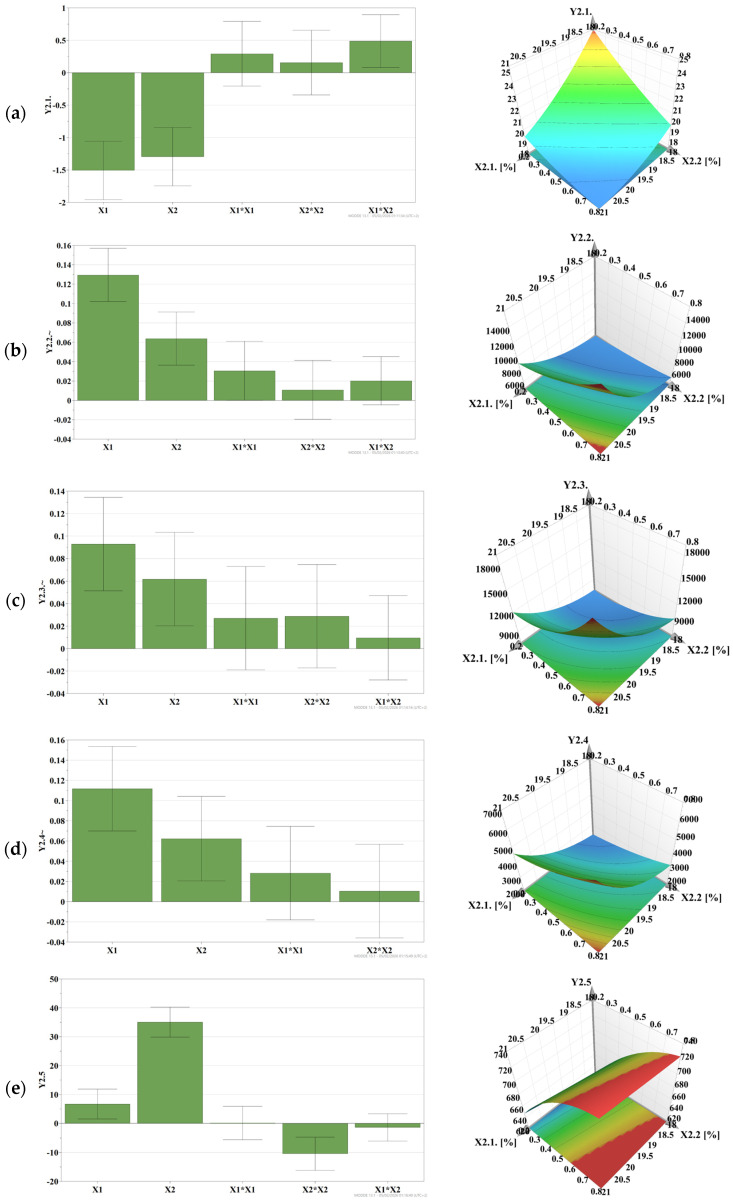

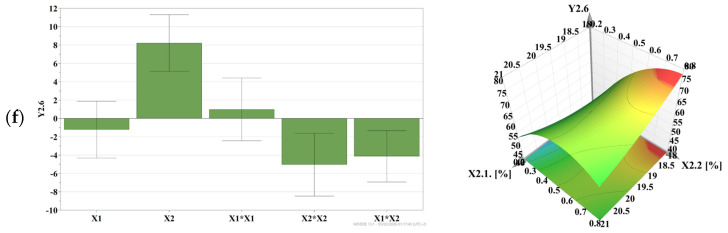
The influence of input variables on: (**a**), spreading (mm)—Y2.1; (**b**), viscosity at 25 °C (mPa·s)—Y2.2; (**c**), viscosity at 37 °C (mPa·s)—Y2.3; (**d**), viscosity of diluted gel at 37 °C (mPa·s)—Y2.4; (**e**), detachment force (mN)—Y2.5; (**f**), bioadhesion force (Pa)—Y2.6.

Figure 3 presents, as histograms, the analysis of centered and scaled coefficients that highlight the influence of formulation factors on the in vitro rheological and adhesion characteristics of MZ-IB-MTBG formulations, consisting of P407 and HPMC K100M:HPMCK4M, 1:1.

##### The Influence of Formulation Factors on In Vitro Rheologic and Adhesive Properties of the Studied MZ-IB-MTBG Formulations

The results showed that the spreading (Y2.1) decreased with increasing polymer concentrations in the formulation, due to the increase in the consistency of the formulations; also, there were P407-HPMC interaction effects, which resulted in an increase in the radius of the spread surface (Figure 3a).

The viscosity of the formulations measured at 25 °C (Y2.2) increased significantly with increasing polymer concentrations in the formulation; nonlinear effects of P407 and P407-HPMC interaction effects were observed, which led to an increase in viscosity at this temperature (Figure 3b). A similar behavior was observed at 37 °C, when the viscosity increased significantly, both in the case of formulations undiluted (Y2.3) and diluted with water (Y2.4), being strongly influenced by the polymer concentration, with P407 having a predominant effect. Nonlinear effects of P407 and HPMC, as well as Poloxamer–HPMC interaction effects on the response, were observed, which determined the increase in viscosity (Figure 3c,d). Also, the viscosity of the studied MZ-IB-MTBG formulations increased after mixing with mucin, the data being used to calculate the bioadhesion force.

The in vitro detachment force (Y2.5) increased significantly with the concentration of HPMC in the formulation, being less influenced by P407. HPMC also showed a nonlinear influence on the response (Figure 3e).

Regarding the bioadhesion force (Y2.6), calculated from the experimental viscosity data, it increased with the concentration of the bioadhesive polymer, HPMC, in the formulation. There was also a nonlinear influence of P407, leading to a reduction in the magnitude of the response. Also, P407—HPMC interaction phenomena were identified (Figure 3f).

The bioadhesion force was calculated starting from the increase in viscosity obtained in the case of MZ-IB-MTBG formulations, measured after they were diluted with the mucin solution; thus, the viscosity obtained was higher than the sum of the viscosities of homologous gels, diluted in the same proportion with water and the viscosity of a mucin dispersion, in the same concentration and at the same temperature. This increase in viscosity, called bioadhesive viscosity (rheological synergism), is due to interactions between the bioadhesive polymers used in the preparation of MZ-IB-MTBG formulations and mucin, and was first described by Hassan and Gallo [31]. Rheological synergism has been proposed as an in vitro parameter to measure the mucoadhesive properties of polymers and is used to calculate bioadhesion force. The higher the rheological synergism, the greater the interaction of the polymer with mucin.

The evaluation of the bioadhesive capacity represents a fundamental stage in the development of a mucoadhesive system and aims to determine the strength of the adhesive bonds between the bioadhesive polymer used in the preparation and the mucus on the surface of the membrane used in the study. The bioadhesive capacity is closely related to the rheological characteristics and increases with increasing viscosity and consistency, provided by a higher concentration of bioadhesive polymers in the formulation, and the results obtained in this study confirm this hypothesis. Data obtained in in vitro or ex vivo studies to determine the detachment force, the strength of interaction with mucin, as well as the evaluation of the time required to remove the formulation from an animal mucosa, can provide valuable information regarding the in vivo performance of the designed product [32].

### 2.3. In Vitro Release Studies

**Figure 4:**
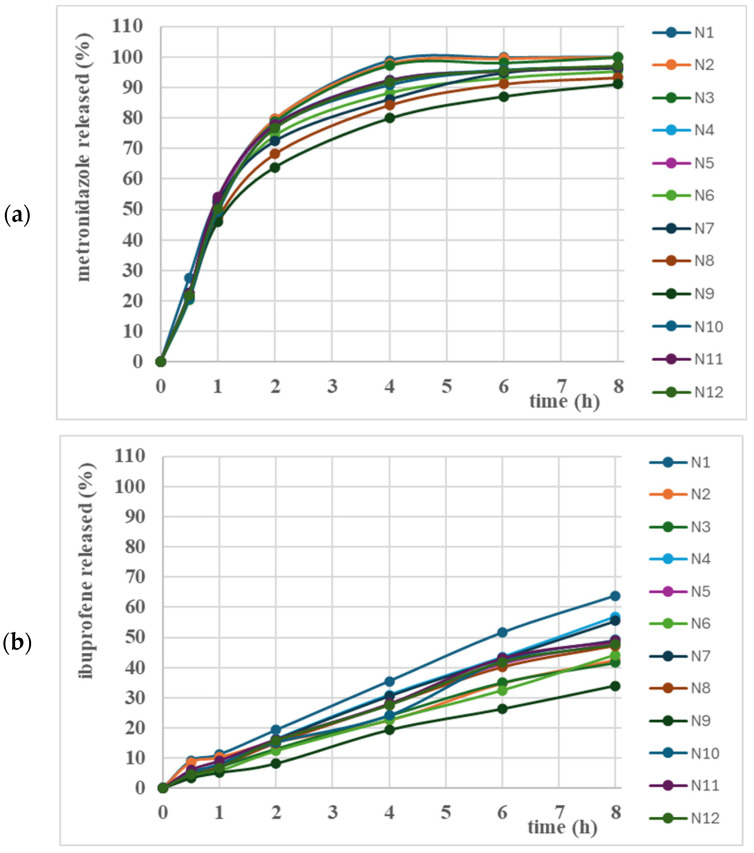
In vitro release of metronidazole (**a**) and ibuprofen (**b**) from mucoadhesive thermosensitive gel based on Poloxamer 407 and HPMC K100M:HPMC K4M, 1:1.

Figure 4 presents the in vitro release profiles of metronidazole (a) and ibuprofen (b). Analysis of the release profiles showed a prolonged release of the two APIs over the 8 h study. MZ was released in a proportion of 80–100%, and IB 30–60%.

The analysis of the release data with several mathematical release models (Baker–Lonsdale, Peppas, Hixon–Crowell, Higuchi, first-order and zero-order equations) led to the conclusion that the best correlation could be achieved with the Peppas model (Tables S3 and S4, Supplementary Files).

**Figure 5:**
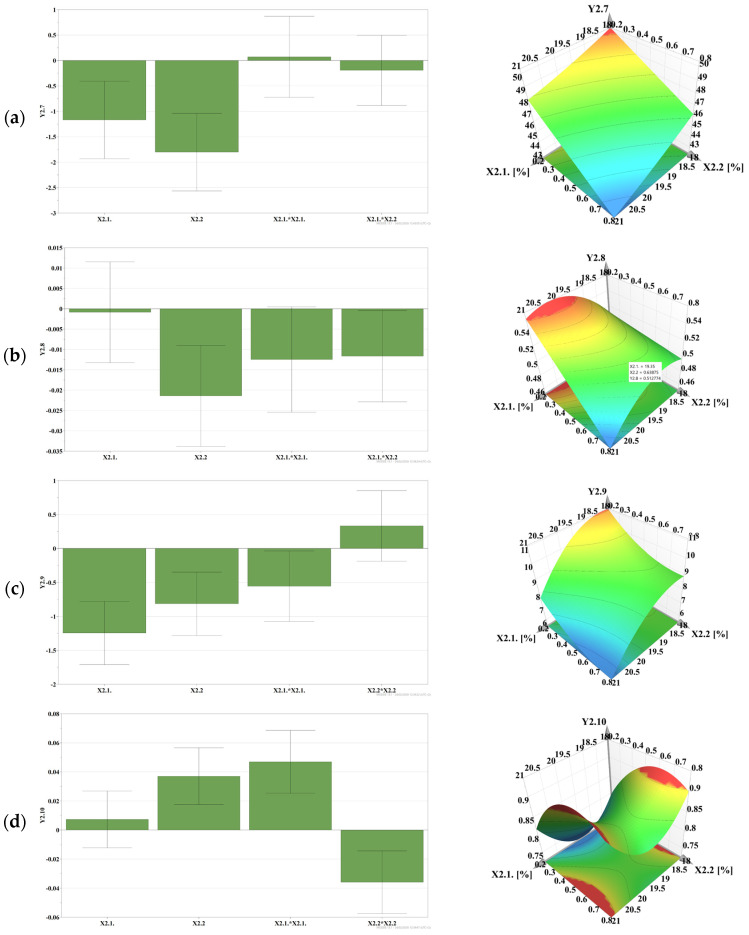
The influence of input variables on: (**a**), *k* Peppas for metronidazole release (h^−n^)—Y2.7; (**b**), *n* Peppas for metronidazole release—Y2.8; (**c**), *k* Peppas for ibuprofen release (h^−n^)—Y2.9; (**d**), *n* Peppas for ibuprofen release—Y2.10, according to optimization DoE.

The analysis of the influence of formulation factors on the release kinetics (Figure 5) showed a negative influence on the release, as the increase in the concentration of polymers in the formulation determined the reduction in the kinetic constant of Peppas’s equation of the two APIs, throughout the entire studied period (Figure 5a,c). These results correlate with those obtained in rheological studies, since the increase in consistency and viscosity reduces the release rate. In the case of IB, the nonlinear influence of P407 is observed, which reduces the release rate, as well as the nonlinear effects of HPMC, which increase the release rate (Figure 5c).

Regarding the release mechanism, in the case of MZ, the *n* values were in the range of 0.4338 and 0.5493, which suggests that the release of MZ occurs by diffusion from the thermosensitive gel matrix. A stronger HPMC influence is observed, increasing the formulation’s consistency and viscosity and, implicitly, reducing the diffusion rate. A nonlinear influence of P407 was also observed, as well as P407-HPMC interactions, which reduced the response (Figure 5b).

Analyzing the release mechanism in the case of IB, the *n* values were between 0.6966 and 0.9367, suggesting a release through combined mechanisms, diffusion–erosion of the gel. HPMC has an effect of increasing response and favoring diffusion; in addition, P407 and HPMC showed nonlinear effects on the release mechanism of IB (Figure 5d). Mechanical and rheological properties are crucial attributes of a gel to ensure the prolonged release of incorporated APIs. Thus, the studied gel formulations containing higher concentrations of HPMCs showed a lower release rate of MZ and IB, as the bioadhesive polymer reduced the erosion rate of P407 and, implicitly, reduced the diffusion rate of APIs [33]. The lower amount and slower release of IB from the studied formulations can be explained by the lower solubility of IB in aqueous medium, compared to MZ. Another explanation could be the occurrence of weak interactions between IB and the excipients used in this study, results obtained in previous studies by DSC/FTIR determinations [34].

### 2.4. Design Space and Optimal Formulations

Risk minimization and establishment of a robust design space represent central objectives of the QbD approach in the development of a thermosensitive buccal mucoadhesive gel. The design space (DS) represents the set of factor combinations that lead to the desired responses (optimal characteristics of a thermosensitive buccal mucoadhesive gel). The design space was constructed by superimposing multiple response surface contour plots and integrating them with probability-based simulations. The likelihood of meeting predefined product specifications within the proposed formulation domain was estimated using the predictive models developed for the identified CQAs in combination with Monte Carlo simulations.

**Figure 6:**
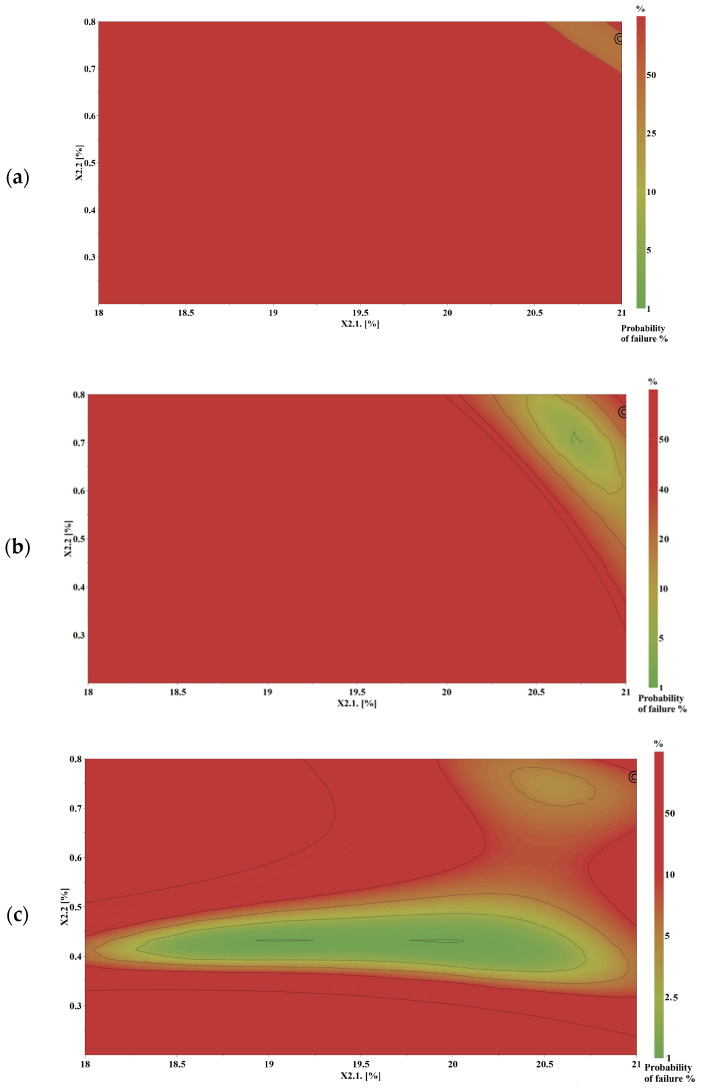
The design space for mucoadhesive thermosensitive buccal gel specifications for viscosity at 37 °C (Y2.3) (**a**), viscosity of diluted gel at 37 °C (Y2.4) (**b**), and bioadhesion force (Pa) (Y2.6.) (**c**); robust setpoint: Poloxamer 407 ratio (X2.1) 20.99, HPMC (blend of K100M:K4M, 1:1) ratio (X2.2) 0.74.

Among the evaluated responses, the most relevant CQAs—such as viscosity at 37 °C (physiological temperature) (Y2.3. and Y2.4.), and bioadhesion force (Y2.6.)—were selected as optimization targets within predefined acceptable intervals in the optimization module of Modde 13.1. software (Table 5). The remaining associated responses were monitored throughout the study to enhance process understanding and to ensure a comprehensive evaluation of the formulation performance and robustness. Based on these settings, the design space for MZ-IB-MTBG formulations was generated (Figure 6).

For the validation of the design space, two formulations were prepared within the DS, with a 5% probability of failure, at the robust setpoint proposed by the software (Robust Point 1: P407 ratio (X2.1) 20.99; HPMC (blend of K100M:K4M, 1:1) ratio (X2.2) 0.74 and Robust Point 2: P407 ratio (X2.1) 19.5; HPMC (blend of K100M:K4M, 1:1) ratio (X2.2) 0.80), and a formulation outside of the DS, with a 50% probability of failure, considered as a negative control: P407 ratio (X2.1) 18.5; HPMC (blend of K100M:K4M, 1:1) ratio (X2.2) 0.20. The residuals of the obtained responses were calculated against the model-predicted values (Table 6), and excellent recovery was observed for both optimized points and negative control. The maximum percentage bias of the formulations prepared under robust point conditions was under 5%, which confirms the validation of the DS, the accuracy of the model equations, and their predictive power.

### 2.5. Additional Characterization of the Optimized Formulation of MZ-IB-MTBG and Stability Studies

#### 2.5.1. Physical Appearance, Gelation Time and Gelation Temperature (Tgel)

**Figure 7:**
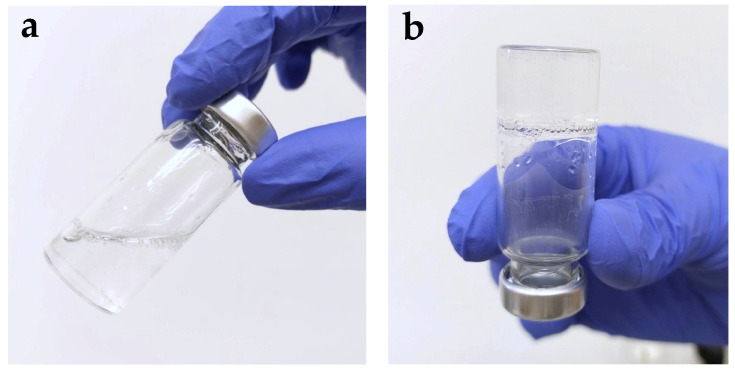

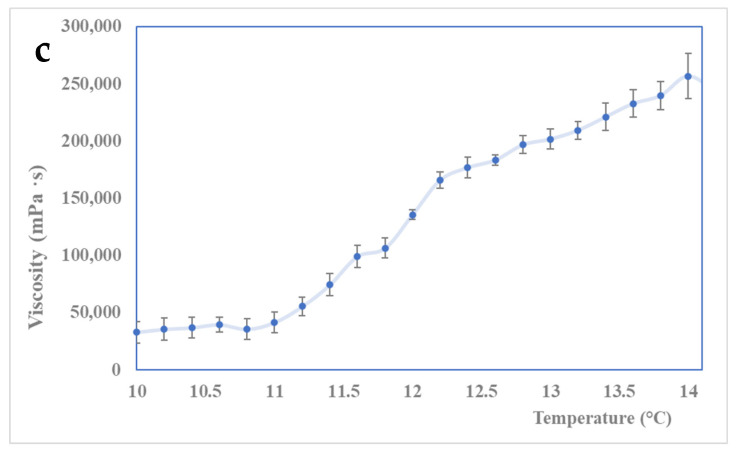
Optimized formulation of MZ-IB-MTBG: (**a**) fluid state formulation; (**b**) completely gelled formulation; (**c**) viscosity–temperature diagram.

The optimized formulation of MZ-IB-MTBG had a homogeneous, transparent-to-slightly-opaque appearance, without particle agglomerations or phase separations when examined with an optical microscope. It is fluid when stored at 2–8 °C (Figure 7a), and by heating to a constant temperature of 37 °C it completely gels within 75–120 ± 11 s (Figure 7b), this state being maintained by storage at room temperature. Studies on the behavior upon increasing temperature identified a Tgel value of approximately 14 ± 0.9 °C (Figure 7c and Table 7).

Although the optimal formulation contains a high concentration of P407, the fluid state at low temperatures enables homogeneous preparation, provided that a low P407 fraction and a low temperature are maintained in the first stage until the APIs are homogeneously dispersed. The temperature-dependent increase in viscosity is a favorable characteristic for the formation of an artificial clot after application in the extraction socket, and the viscous gel state allows for good stability during the storage period [35,36,37].

#### 2.5.2. Viscosity

The viscosity of the optimized formulation was studied under the conditions provided in the experimental plan, and special interest was given to the behavior at 37 °C, which decisively influences the residence time on the mucosa. The determination of the flow curve after preparation revealed a good correlation with the Power Law equation, with values of flow index (*n*) of 0.04 (Figure S1), which means a pseudoplastic flow. In addition, to evaluate the physical stability of the optimized MZ-IB-MTBG formulation during preservation, the viscosity was studied at low rotation speeds (10 s^−1^), at a preservation temperature of 25 °C, and the values obtained at 6 months and 12 months after preparation did not show statistically significant differences (*p* > 0.05) (Table 7).

#### 2.5.3. Drug Content

The uniformity of the API content of the MZ-IB-MTBG optimized formulation was tested, and the values identified were over 90% for both drug substances, at preparation and after 6 months and 12 months of storage. These results confirm the homogeneity of the gel formulation, as well as the physicochemical stability of the APIs incorporated in the vehicle consisting of a mixture of HPMCs and P407, under the established storage conditions (Table 7).

#### 2.5.4. pH

The pH measurement provides information about the tolerance of the gel on the oral mucosa, since a pH value far from the physiological one can be irritating. The optimal formulation presented values between 5.95 ± 0.22 and 6.2 ± 0.45 (Table 7), which can be considered as having good tolerance.

#### 2.5.5. Ex Vivo Mucoadhesion Time

The average time required for the detachment of the optimized MZ-IB-MTBG formulation from the chicken esophageal mucosa was 192 ± 25 min (Table 7), which highlights suitable adhesion capacity after application in the extraction socket.

Ex vivo residence time studies of mucoadhesive gels are critical for predicting how long a formulation will remain attached to human oral tissue, such as the buccal mucosa, gingiva, or tongue, before being washed away by saliva or removed by mechanical action (tongue movement, swallowing, chewing). These studies use fresh excised animal tissue to measure the adhesion strength and endurance of the gel [32,38].

#### 2.5.6. In Vivo Evaluation

The pilot exploratory study included 15 patientswho considered at high risk of developing post-extraction alveolar osteitis. Due to the high-risk profile of the included patients, withholding local prophylactic treatment was considered ethically inappropriate. Therefore, the study was designed as a single-arm exploratory evaluation focused on safety, feasibility, and preliminary clinical performance. The selected group was predominantly female (10/15 patients), with a mean age of 42.3 ± 13.5 years (range: 28–78 years). Among the systemic risk factors identified, type 2 diabetes mellitus was the most common (seven cases), followed by oral contraceptive use, active smoking, and other associated local or general conditions. Most patients included in the study were active smokers (11/15), confirming the high-risk nature of the population, and 86.7% of them had poor or average oral hygiene, confirming an increased risk for post-extraction complications (alveolar osteitis). The general level of oral hygiene is correlated with the occurrence of alveolar inflammatory complications. Postoperative pain assessment 5–7 days after extraction revealed a reduced level of pain symptoms, with a mean pain score of 1.4 (range: 1–3). Most patients reported minimal discomfort (score 1), and only one patient had a score of 3. The clinical diagnosis of alveolar osteitis was based on established criteria, including the presence of increasing postoperative pain between days 3 and 7, exposed alveolar bone, and/or associated fetid odor or local inflammatory signs. The absence of these clinical features, together with the presence of a stable clot or normal granulation tissue, was considered indicative of favorable healing. No cases of clinically manifest alveolar osteitis were recorded during the follow-up period.

The in vivo persistence of the antiseptic and anti-inflammatory mucoadhesive gel in the post-extraction socket was assessed as satisfactory, with a retention time of approximately 3 to 6 h, most frequently in the range of 4–5 h. No local adverse reactions were observed; specifically, no mucosal irritation or inflammatory reactions secondary to the application of the product were reported. Bitter taste scores were consistently maximum on the scale used, but without causing discontinuation of use or additional complaints from patients. From a clinical point of view, the postoperative evolution was favorable in all cases evaluated, without the occurrence of local inflammatory complications, with satisfactory tissue healing and the absence of clinical signs suggestive of clot disintegration or alveolar infection.

### 2.6. Limitations and Future Research Directions

The research presents certain limitations that may represent the objective of further studies, regarding the absence of stability studies according to ICH Q1A, which consist in the identification over time of possible impurity profiles in the optimized MZ-IB-MTBG formulation.

Also, the present in vivo evaluation should be interpreted in the context of its exploratory design. The clinical component was conducted as a pilot, single-arm study aimed primarily at assessing the feasibility, local tolerance, retention, and preliminary clinical performance of the developed mucoadhesive thermosensitive gel in a population at increased risk of alveolar osteitis. The relatively small sample size and the absence of a control group represent inherent limitations of this approach; however, withholding local prophylactic treatment in high-risk patients was considered ethically inappropriate, as the use of intra-alveolar dressings with antiseptic, antimicrobial, or anti-inflammatory effects is consistent with routine clinical practice. The follow-up interval of 5–7 days was selected based on the well-established timeframe for the onset of alveolar osteitis, allowing the detection of early postoperative complications, although it does not capture longer-term healing dynamics. Therefore, the results of this preliminary evaluation should be interpreted with caution. Future studies are warranted to further validate these findings through larger patient cohorts, controlled comparative designs, and extended follow-up periods, to more comprehensively assess both clinical efficacy and long-term outcomes.

## 3. Conclusions

An optimized formulation of MZ-IB-MTBG was prepared using the QbD approach and is designed for application in the oral cavity. The preparation method allowed for obtaining homogeneity, an appropriate pH, and in vitro characterization revealed a pseudoplastic behavior, which allows easy, non-invasive application. The formulation showed an increase in viscosity at 37 °C, indicating the ability to form a stable artificial clot after application in the socket. In vitro adhesion studies identified the ability to interact with mucin, and the ex vivo mucoadhesion time had promising values. The in vitro release of MZ and IB was prolonged, and the kinetics were correlated with the Peppas model. Studies carried out over 12 months revealed a concentration of over 90% of MZ and IB, with insignificant variation in viscosity; these results confirm the stability of the gel and validate the preparation method and storage conditions (25 °C).

In vivo evaluation after application in the extraction socket revealed favorable postoperative evolution, with satisfactory tissue healing and a reduced level of pain symptoms. In vivo, residence on the socket was satisfactory, and no local discomfort was reported.

The optimized formulation of MZ-IB-MTBG may represent a promising platform for drug delivery in the oral cavity and could serve as a clot supplement to prevent alveolar osteitis. The developed vehicle, with adequate mechanical and adhesive properties, may incorporate different APIs, depending on clinical requirements, and the application may have other destinations (gum, cheek), but with other challenges regarding residence time, which would be the subject of further studies.

## 4. Materials and Methods

### 4.1. Materials

Poloxamer P 407 (Kolliphor P407) was kindly obtained from BASF (Ludwigshafen, Germany), HPMC K100M (Methocel K100M Premium) and HPMC K4M (Methocel K4M Premium) were a generous gift from Colorcon Limited (Dartford, UK), ibuprofen and metronidazole were from BASF (Ludwigshafen, Germany), polyethylene glycol PEG 400 was from Merck (Darmstadt, Germany). Polyethylene oxide (PEO, POLYOX™ WSR 1105) was kindly provided by Dow Chemical (Midland, MI, USA). Mucin from porcine stomach, Type III, was from Sigma-Aldrich (Darmstadt, Germany). All other reagents were of analytical grade.

### 4.2. Preparation of Thermosensitive Mucoadhesive Gel Systems

In the first step, HPMCs or PEO1105 were dispersed in pre-heated distilled water and mixed with P407 previously dispersed in cold water. For dispersion and deaeration, the formulations were kept at 4 °C for 12 h, then gently homogenized and stored at room temperature in closed containers. In the case of MZ-IB-MBTG formulations, metronidazole was initially added to the gel system at a reduced concentration of P407 and kept under continuous stirring for 30 min at 10–12 °C. Ibuprofen was incorporated as a PEG 400 solution. The samples were stored for 24 h to achieve stabilization and were further subjected to characterization. The optimized formulation was stored in brown glass containers and was tested over time (6 months and 12 months) to evaluate the stability of the system.

### 4.3. The QbD Approach

#### 4.3.1. Risk Analysis

The Quality Target Product Profile (QTPP) for the mucoadhesive, thermosensitive buccal gel was established initially, and the corresponding critical quality attributes (CQAs) were subsequently determined.

According to ICH Q8(R2) and ICH Q9 guidelines, the prospective use of quality risk management tools is recommended for the identification of independent variables (critical material attributes and critical process parameters) before conducting experimental studies [29].

To support risk evaluation, Ishikawa (fishbone) diagrams were constructed to systematically examine formulation-, process-, characterization-, and methodology-related factors that could influence product quality. Failure Mode and Effects Analysis (FMEA) was employed as a risk management tool to assess and rank variables that could impact product performance. For each identified critical factor, a Risk Priority Number (RPN) was derived by multiplying the assigned scores for occurrence (O), severity (S), and detectability (D) of possible failure modes. The probability of occurrence was rated on a five-point scale: 5 (frequent), 4 (probable), 3 (occasional), 2 (remote), and 1 (unlikely). Severity, reflecting the impact of a failure, was categorized as 5 (catastrophic), 4 (critical), 3 (serious), 2 (minor), and 1 (negligible). Detectability was also rated from 1 to 5, where 1 indicated failures that are easy to detect, 2 indicated high detectability, 3 indicated moderate detectability, 4 indicated a low likelihood of detection, and 5 indicated failures that are difficult to detect.

Parameters associated with elevated RPN values were controlled by further investigating their impact through a Design of Experiments (DoE)-guided development.

#### 4.3.2. Design of Experiments

For screening purposes, a D-optimal experimental design with 21 runs and three factors was generated (Table 8). In these formulations, the first formulation factor (X1.1) was P407 in different concentrations, 12–15–18%. The second formulation factor (X1.2.) was used at concentrations of 1–2–3%, in combination with P407, and the third formulation factor was the nature of the second polymer: PEO grade 1105 or HPMC grade K100M. The dependent variables (Y-responses) evaluated were spreadability, viscosity, and detachment force (Table 8). Data fitting and the calculation of statistical parameters to validate the experimental plan were performed using the Modde 13.1 optimization program (Sartorius Stedim, Malmö, Sweden). Data fitting was performed using Partial Least Squares (PLS).

For optimization purposes, 12 runs of a full-factorial experimental design with two factors at three levels each were generated (Table 9). In these formulations, P407 in different concentrations (X2.1), 18-19.5-21%, was combined with different concentrations of hydroxypropyl methylcellulose—HPMC (X2.2), 0.2–0.5–0.8%. HPMC was used as a mixture of two grades, K100M: K4M, in 1:1 ratio. The dependent variables (Y-responses) evaluated were represented by the rheological, in vitro adhesion, and dissolution properties (Table 9). The data fitting and the calculation of statistical parameters for validating the experimental plan were performed similarly to those in the previous DoE.

### 4.4. Determination of Pharmaceutical Characteristics of Gel Bases and MZ-IB-MTBG Formulations Used as DoE Responses

#### 4.4.1. Study of Spreading Capacity

Spreadability was determined by measuring the radius of the spreading surface (mm) occupied by 1 g of formulation placed between two 20 × 20 cm glass plates [39]. The mass of the upper plate was standardized to 68 g, and an additional 500 g mass was placed on the device. Spreadability was determined after 1 min, and the results represent the average of three determinations.

#### 4.4.2. Viscosity Studies

The viscosity measurements were performed on a Brookfield DV3T cone and plate viscosimeter (Brookfield Engineering Laboratories, Inc., Middleboro, MA, USA), with three replicates for each experiment. Since viscosity decreases with increasing rotation speed, to compare gel formulations, the apparent viscosity at 20.38 s^−1^ was considered. The MZ-IB-MTBG formulations were studied at 25 °C ± 0.5, as well as at 37 °C ± 0.5, and at this temperature, measurements were madeon the undiluted formulations and after dilution in a ratio of 20% with water, or mucin solution (4% mucin in gel).

In addition, to optimize the formulation, the viscosity was determined at a low rotation speed (10 s^−1^) and a storage temperature of 25 °C to verify stability at 6 and 12 months after preparation. The flow curve was also plotted (in the range of 1.22–64.82 s^−1^), and the data were fitted to the Power Law mathematical model, where:

(1) $$\tau =K\cdot \gamma^{n}$$

where:

τ—shear stress (Pa);
*γ*—shear rate (s^−1^);
*n*—the non-Newtonian index (0 < *n* < 1);
K—consistency index (a factor related to the apparent viscosity of the gel) [40,41].

#### 4.4.3. The Study of In Vitro Bioadhesion Properties

##### The In Vitro Detachment Force

The in vitro detachment force was determined at 25 ± 1 °C, by measuring the necessary mass to detach a synthetic membrane (cellulose), immersed in a 1% aqueous mucin dispersion, brought into contact with the studied sample, and calculated according to the formula:

(2) F=m·g

where:

*F*—the detachment force (mN)
*m*—maximum weight needed for detachment (g)
*g*—gravitational acceleration (m·s^−2^)

An experimental device was used (balance Mechaniki precyzyincj Zaklady, Poland), adapted according to the literature [42]. The results represent an average of seven determinations.

##### Bioadhesion Force

Bioadhesion force was calculated by measuring the viscosity of MZ-IB-MTBG formulations diluted in 20% water and with a mucin solution, according to the theory proposed by Hassan and Gallo [31,43]. The method has important practical implications, as it allows the evaluation of the bioadhesive potential of the formulation. The viscous component of bioadhesion (rheological synergism) (*η_b_*) was calculated using the equation:

(3) $$\eta_{t}=\eta_{m}+\eta_{p}+\eta_{b}$$

where:

$\eta_{t}$—system viscosity;
$\eta_{m}$—mucin viscosity;
$\eta_{p}$—bioadhesive gel viscosity;
$\eta_{b}$—rheological synergism.

The bioadhesion force was calculated using the following equation:

(4) $$F=\eta_{b}\gamma$$

where

γ—deformation speed (s^−1^);
$\eta_{b}$—rheological synergism, an empirical determinant of the absolute bioadhesion force, calculated using experimental values measured under identical conditions of concentration, temperature, time, and rotation speed. This parameter also reflects different physicochemical properties of bioadhesive polymers, such as molecular weight, electrostatic charges, and configuration.

#### 4.4.4. In Vitro Dissolution Testing

Dissolution testing was carried out using a Pharma Test PT-DT7 (Pharma Test, Hainburg, Germany) dissolution apparatus in phosphate buffer, pH 6.8, by placing 1–2 g of mucoadhesive gel in dialysis bags (Spectra/Por Cellulose Ester Membrane MWCO: 5000–8000 Da, Repligen, Waltham, MA, USA). The dialysis bags were sealed with special clamps and immersed in the receiving medium, which was maintained at 37 °C under continuous stirring using paddles at a speed of 50 rpm. A total of 5 mL aliquots was sampled every 30 min, 1 h, 2 h, 4 h, 6 h, and 8 h, each time replacing the sampled volume with fresh media to maintain constant dissolution conditions [42]. Metronidazole and ibuprofen were quantified using a validated HPLC-UV method using an Agilent 1100 series apparatus (Agilent Inc., Santa Clara, CA, USA). Chromatographic separation was performed on a Zorbax SC C18 column (5 μm, 4.6 × 150 mm), and detection was performed at 319 nm for metronidazole and 225 nm for ibuprofen. The mobile phase was acetonitrile and 0.1% phosphoric acid (15:85 *v*/*v*) with a flow rate of 1 mL/min. Under those chromatographic conditions, the retention times for ibuprofen and metronidazole were 2.12 min and 2.48 min, respectively (the method was validated for specificity, linearity (y = 39.007x − 18.373, R = 996), accuracy 95.64–102.84%, and precision RSD 3.73%, in a range of 3–18 µg/mL for ibuprofen and specificity, linearity (y = 34.432X − 4.711, R = 994), accuracy 97.97–102.48%, and precision RSD 2.11%, in a range of 3–18 µg/mL for metronidazole). The experiments were repeated 3 times; the mean values and standard deviations were calculated.

#### 4.4.5. Kinetic Release Estimation

Kinetic release of Mz and IB from the prepared formulations was evaluated by fitting different mathematical models (Table 10) to the release profiles [44,45]. Only values higher than 80% were considered in the calculation of release kinetics.

#### 4.4.6. Additional Characterization of the Optimized MZ-IB-MTBG Formulation

##### Homogeneity

For homogeneity determination, microscopic images were obtained using a Zeiss microscope at 4× magnification (scale 500 µm).

##### Gelation Temperature (Tgel) and Gelation Time

The gelation temperature, Tgel, was determined for the optimized formulation, which was fluidized in the refrigerator. The viscosity was determined as a function of temperature in the range of 10 °C to 15 °C at a rotation speed of 10 s^−1^, with the temperature increased in increments of 0.2 °C [46]. To study the gelation time, the optimized MZ-IB-MTBG formulation was maintained at a constant temperature of 37 °C ± 0.5 °C. The time interval required for complete gelation was measured. The results represent the average of three determinations.

##### Uniformity of Drug Content

Gel samples were taken from three different areas, weighed, and then dispersed in methanol. After centrifugation, MZ and IB were quantified by HPLC methods described previously.

##### pH Determination

The pH of the optimized MZ-IB-MTBG formulation was measured by dispersing 1 g of the gel sample in 100 mL of water (using a calibrated Mettler Toledo MP 225 digital pH meter). The results represent the average of three determinations [47,48].

##### Ex Vivo Mucoadhesion Time

The time required for the optimized formulation to detach from a natural membrane was determined according to a method described in the literature [14,49]. Fresh chicken esophageal mucosa was used, fixed with cyanoacrylate on a double-sided adhesive tape on a Plexiglas plate, and placed in an adapted tablet disintegrator (Erveka GmbH, Langen, Germany). An amount of 1 mL of gel was applied to the mucosa and spread with a spatula so that the gel occupied the surface of a 4 × 2.5 cm rectangle. The gel was repeatedly immersed in a phosphate buffer solution at pH 6.8 and 37 ± 1 °C until complete elution. The results represented the average of five determinations.

##### In Vivo Evaluation

The experimental protocol for the clinical evaluation of the optimized MZ-IB-MTBG formulation was approved by the Ethics Committee of the “Iuliu Hațieganu” University of Medicine and Pharmacy, Cluj-Napoca (Approval No. 32/25 February 2019). Prior to mucoadhesive formulation application, all patients received detailed information about the study protocol and procedures, and written informed consent was obtained from each participant. All procedures were conducted in accordance with the ethical standards outlined in the Declaration of Helsinki for research involving human subjects, as well as applicable national and European Union regulations.

The inclusion criteria in the study were represented by the risk of alveolar osteitis and the presence of at least two predisposing factors: active smoker, poor hygiene, and controlled type 2 diabetes. The exclusion criteria for the study were: uncontrolled chronic diseases, a history of antiresorptive or antiangiogenic treatment, and a history of radiotherapy. Preoperative oral hygiene was assessed on a scale of 1 to 3, with 1 indicating low and 3 indicating moderate levels. Most patients (46.7%) had poor oral hygiene (score 1), while 13.3% had good oral hygiene (score 3). The average oral hygiene score for the entire group (1.67) suggests a modest level of plaque control in the studied population.

The optimized MZ-IB-MTBG formulation, containing 1% MZ and IB and prepared under aseptic conditions, was applied to the extraction socket. Patients were advised to avoid solid foods, rinse their mouths, and spit for 6 h. The healing of the post-extraction dental alveolus was evaluated in patients at increased risk of alveolar osteitis, to whom a formulation with antiseptic and anti-inflammatory action was applied prophylactically.

## Figures and Tables

**Table 1:** The summary of QTPP and CQAs of mucoadhesive thermosensitive buccal gel formulation.

QTPP Elements		Target	Is This a CQA?	Justification
Dosage form		Gel	No	
Route of administration		Buccal	No	
Drug content		1.00% (*w*/*w*) metronidazole 1.00% (*w*/*w*) ibuprofen	No	Content uniformity is directly linked with homogeneity and affects safety and efficacy
Product quality attributes	Appearance	Homogeneous	No	Indicates a homogeneous, physically stable gel, allowing uniform distribution of APIs
	Identification	Positive for metronidazole and ibuprofen	No	API identification is critical for safety and efficacy. However, this feature will be monitored at drug product release
	API content after 6 and 12 months	90% to 110% of the labeled content	No	Recovery of the drugs after 12 months of storage is directly linked to their stability and affect safety and efficacy
	Spreadability (mm)	17–19	Yes	Spreading can affect the local application of the gel
	Viscosity at 25 °C and 20.38 s^−1^ (mPa·s)	11,000–15,000	Yes	May affect gel application and adhesion to the mucosa
	Viscosity at 37 °C and 20.38 s^−1^ (mPa·s)	13,000–18,000	Yes	A value higher than the value at 25 °C directly influences the formation of an adherent film on the mucosal surface
	Viscosity of diluted gel with water at 37 °C and 20.38 s^−1^ (mPa·s)	5200–5900	Yes	Viscosity directly influences application and adhesion to the mucosa
	Detachment force (mN)	649–735	Yes	Indicates the ability to adhere to the mucosa, with an effect on therapeutic efficacy
	Bioadhesion force (Pa)	60–74	Yes	Directly estimates the gel–mucin interaction that occurs in the mucoadhesion process
	Dissolution at pH 6.8	Metronidazole release: Not less than 20% at 0.5 h Not less than 60% at 2 h Not less than 90% at 6 h	Yes	Indicates the local release of APIs in effective concentration, during the residence time of the gel on the mucosa
		Ibuprofen release: Not less than 5% at 0.5 h Not less than 15% at 2 h Not less than 40% at 6 h	Yes	
	pH	No less than 5.5	No	Affects the tolerance of the gel on the oral mucosa
	Ex vivo mucoadhesion time (min)	No less than 60 min	No	Estimates the residence time on the mucosa and affects therapeutic efficacy

**Table 2:** Failure Mode and Effects Analysis.

Nr	CMC/CPP	Failure Mode	Failure Effects	Potential Causes	Control Methods	O	S	D	RPN
1	Polymer type	Lack of bioadhesive properties; improper rheological properties	Low residence time and poor adhesion	Inappropriate physical and chemical characteristics	DoE-based formulation optimization	5	5	3	75
2	Polymer concentration			Unsuitable concentration		5	5	3	75
3	Polymer molecular weight			Inappropriate physical and chemical characteristics		5	5	3	75
4	Polymer gelation temperature			Inappropriate physical and chemical characteristics		5	5	3	75
5	API concentration	Lack of homogeneity; variable/incomplete dissolution	Reduced therapeutic effect	API remains partially undissolved	Evaluate API solubility	3	3	3	27
6	API solubility				Use excipients that favor dissolution	3	4	3	36
7	Process temperature	Lack of homogeneity	Low residence time, poor adhesion, variable dissolution, incomplete polymer hydration, non-uniform viscosity, dose variability	The formulation components are not homogeneously distributed in the structure of the gel	Ensure appropriate process conditions for optimal ingredient mixing and complete polymer hydration; standardize the preparation procedure	3	3	3	27
8	Process hydration time					3	3	3	27
9	Process stirring speed					3	2	3	18
10	Process addition order					2	4	3	24
11	Process time					2	4	3	24
12	Characterization temperature	Improper testing conditions; incorrect estimation of product performance	Optimization performed on data that is not relevant; variable drug release	Variable testing conditions; non-representative testing conditions	Use relevant testing conditions that are representative of in vivo administration	2	3	3	18
13	Dissolution media type					2	3	3	18
14	Dissolution pH					2	3	3	18

O—occurrence; S—severity; D—detectability. RPN—Risk Priority Number (RPN=O·S·D).

**Table 3:** Response matrix and quality-of-fit parameters of screening DoE.

Exp Name	Run Order	Y1.1	Y1.2	Y1.3
N1	10	42.0	7176	26.0
N2	4	28.0	28,996	120.0
N3	13	39.0	20,803	38.0
N4	1	22.0	49,802	200.0
N5	21	39.5	17,363	29.0
N6	16	24.0	41,976	180.0
N7	7	38.0	22,919	120.0
N8	20	33.0	22,980	170.0
N9	2	37.0	20,624	165.0
N10	18	33.5	17,018	145.0
N11	12	37.5	22,136	162.0
N12	19	40.0	2943	28.0
N13	5	27.0	32,808	210.0
N14	15	37.0	15,081	35.0
N15	3	20.5	52,184	250.0
N16	17	38.0	13,626	30.0
N17	9	22.5	43,540	220.0
N18	11	35.0	19,738	110.0
N19	14	32.0	24,272	190.0
N20	8	34.0	20,108	140.0
N21	6	32.0	16,923	118.0
Statistical parameter				
Goodness of fit, R^2^		0.928	0.874	0.896
R^2^ adjusted		0.909	0.832	0.861
Goodness of prediction, Q^2^		0.891	0.708	0.799
Model validity		0.862	0.413	0.411
Reproducibility		0.910	0.963	0.969

Spreadability (mm)—Y1.1; viscosity at 37 °C (mPa·s)—Y1.2; detachment force (mN)—Y1.3.

**Table 4:** Response matrix and quality-of-fit parameters for optimization DoE.

Exp Name	Run Order	Y2.1	Y2.2	Y2.3	Y2.4	Y2.5	Y2.6	Y2.7	Y2.8	Y2.9	Y2.10
N1	6	25.23	4900	6700	2320	622	40.42	50.557	0.5121	11.636	0.8198
N2	1	21.94	5180	8990	2455	633	40.72	47.978	0.5493	9.745	0.6996
N3	2	19.12	9660	12,100	5290	649	56.06	47.827	0.5469	7.668	0.8252
N4	5	22.35	5390	8925	2870	701	72.51	47.829	0.5141	8.756	0.8993
N5	10	19.75	6720	9050	3360	705	65.80	47.868	0.5110	8.737	0.8357
N6	12	18.75	11,060	13,230	5320	711	61.13	45.211	0.5231	6.219	0.9367
N7	7	19.87	5550	9030	3120	718	74.13	46.409	0.4846	8.652	0.8963
N8	9	18.56	9450	13,370	5200	734	68.24	43.058	0.5158	8.700	0.8265
N9	3	17.34	15,400	19,180	5950	735	60.12	42.249	0.4338	5.152	0.9105
N10	4	19.9	6910	9200	3345	706	65.80	45.663	0.5376	8.007	0.8838
N11	8	20.1	6880	8990	3211	702	69.03	48.289	0.5080	9.670	0.7954
N12	11	19.5	6734	9300	3375	705	65.91	46.432	0.5302	8.691	0.8377
Statistical parameter											
Goodness of fit, R^2^		0.956	0.968	0.893	0.888	0.981	0.921	0.865	0.800	0.901	0.883
R^2^ adjusted		0.919	0.940	0.804	0.824	0.965	0.855	0.788	0.686	0.844	0.816
Goodness of prediction, Q^2^		0.540	0.559	0.513	0.600	0.604	0.578	0.517	0.548	0.582	0.747
Model validity		0.928	0.420	0.715	0.301	0.857	0.580	0.911	0.724	0.860	0.971
Reproducibility		0.886	0.982	0.860	0.964	0.961	0.930	0.722	0.783	0.831	0.680

Spreadability (mm)—Y2.1; viscosity at 25 °C (mPa·s)—Y2.2; viscosity at 37 °C (mPa·s)—Y2.3; viscosity of diluted gel at 37 °C (mPa·s)—Y2.4; detachment force (mN)—Y2.5; bioadhesion force (Pa)—Y2.6; *k* Peppas for metronidazole release (h^−n^)—Y2.7; *n* Peppas for metronidazole release—Y2.8; *k* Peppas for ibuprofen release (h^−n^)—Y2.9; *n* Peppas for ibuprofen release—Y2.10.

**Table 5:** Optimization criteria.

Responses	Objective	Minimum	Target	Maximum
Spreadability (Y2.1)	Predicted	-	-	-
Viscosity at 25 °C (Y2.2)	Predicted	-	-	-
Viscosity at 37 °C (Y2.3)	Target	16,000	18,000	19,100
Viscosity of diluted gel at 37 °C (Y2.4)	Target	4800	5800	6800
Detachment force (Y2.5)	Predicted	-	-	-
Bioadhesion force (Y2.6)	Target	55	62	67
*k* Peppas for metronidazole release (Y2.7)	Predicted	-	-	-
*n* Peppas for metronidazole release (Y2.8)	Predicted	-	-	-
*k* Peppas for ibuprofen release (Y2.9)	Predicted	-	-	-
*n* Peppas for ibuprofen release (Y2.10)	Predicted	-	-	-

**Table 6:** Validation results of design space at the robust setpoint.

	Optimization 1—Robust Point (RP1)				Optimization 2—Robust Point				Negative Control (NC)			
Responses	Predicted	Experimental	Residual	%Bias	Predicted	Experimental	Residual	%Bias	Predicted	Experimental	Residual	%Bias
Spreadability (Y2.1)	17.71	17.58 ± 2.35	−0.130	−0.734	18.04	17.21 ± 2.82	−1.028	−4.590	23.94	25.56 ± 1.86	1.618	6.757
Viscosity at 25 °C (Y2.2)	14,461.50	13,998 ± 760	−463.50	−3.205	8564.45	8375 ± 707	−189.45	−2.212	4927.54	4978 ± 335	50.46	1.024
Viscosity at 37 °C (Y2.3)	17,552.60	17,998 ± 586	445.40	2.538	12,256.50	12,480 ± 675	223.50	1.824	7429.13	6790 ± 135	−639.13	−8.603
Viscosity of diluted gel at 37 °C (Y2.4)	5861.46	5725 ± 334	−136.46	−2.328	4245.23	4402 ± 287	156.77	3.693	2405.43	2340 ± 89	−65.43	−2.720
Detachment force (Y2.5)	733.23	745.6 ± 45	12.37	1.687	729.92	721 ± 67	−8.923	−1.222	627.37	635 ± 21	7.626	1.216
Bioadhesion force (Y2.6)	62.22	61.92 ± 4.55	−0.300	−0.482	66.93	67.11 ± 5.53	0.183	0.273	41.53	42.67 ± 8.90	1.143	2.752
*k* Peppas for metronidazole release (Y2.7)	43.98	42.53 + 6.64	−1.450	−3.297	44.11	42.81 ± 7.22	−1.297	−2.941	49.87	51.54 ± 1.21	1.674	3.357
*n* Peppas for metronidazole release (Y2.8)	0.493	0.479 ± 0.007	−0.014	−2.840	0.501	0.522 ± 0.009	0.021	4.163	0.531	0.513 ± 0.008	−0.018	−3.330
*k* Peppas for ibuprofen release (Y2.9)	7.507	7.345 ± 0.25	−0.162	−2.158	8.18	8.55 ±0.15	0.368	4.497	11.06	10.95 ± 1.09	−0.107	−0.964
*n* Peppas for ibuprofen release (Y2.10)	0.847	0.830 ± 0.004	−0.017	−2.007	0.821	0.808 ± 0.0045	−0.013	−1.622	0.753	0.825 ± 0.0067	0.072	9.598

**Table 7:** Composition and physicochemical characteristics of optimized MZ-IB-MTBG formulation.

**Composition**	**% (g)**	**mg/g Gel**	
Poloxamer 407 ratio	20.99	209.90	
HPMC *K100M*	0.37	3.70	
HPMC *K4M*	0.37	3.70	
Ibuprofen	1	10	
Metronidazole	1	10	
Water	76.27	762.70	
**Physicochemical Characteristics**	**Fresh**	**6 Months After** **Preparation**	**12 Months After Preparation**
Appearance	Transparent to slightly opaque	Transparent to slightly opaque	Transparent to slightly opaque
pH	6.2 ± 0.45	6.1 ± 0.24	5.95 ± 0.31
T_gel_ (°C)	14 ± 0.9	-	-
Gelation time (s)	75–120 ± 11	-	-
Recovery in MZ content (%)	98.20 ± 0.45	96.35 ± 0.21	94.67 ± 0.27
Recovery in IB content (%)	97.53 ± 0.89	97.11 ± 1.23	95.42 ± 1.98
Viscosity at 10 s^−1^ (mPa·s)	32,901 ± 302	33,420 ± 267	34,096 ± 330
Ex vivo bioadhesion time (min)	192 ± 25	-	-

**Table 8:** D-optimal DoE experimental design for screening.

**Independent Variables**					**DoE Matrix**			
**Formulation Factors**	**Symbol**	**Levels of Variation**			**Exp**	**X1.1**	**X1.2**	**X1.3**
		**−1**	**0**	**+1**				
Polymer 1 * ratio	X1.1	12	15	18	N1	12	1	PEO
Polymer 2 ratio	X1.2	1	2	3	N2	18	1	PEO
Polymer 2 type	X1.3	PEO **		HPMC ***	N3	12	3	PEO
**Dependent Variables**					N4	18	3	PEO
					N5	12	2	PEO
					N6	18	2	PEO
**Responses**			**Symbols**		N7	15	1	PEO
Spreadability (mm)			Y1.1		N8	15	3	PEO
Viscosity at 37 °C (mPa·s)			Y1.2		N9	15	2	PEO
Detachment force (mN)			Y1.3		N10	15	2	PEO
* Poloxamer 407					N11	15	2	PEO
** PEO grade 1105					N12	12	1	HPMC
*** HPMC grade K100M					N13	18	1	HPMC
					N14	12	3	HPMC
					N15	18	3	HPMC
					N16	12	2	HPMC
					N17	18	2	HPMC
					N18	15	1	HPMC
					N19	15	3	HPMC
					N20	15	2	HPMC
					N21	15	2	HPMC

**Table 9:** Full factorial DoE for optimization.

**Independent Variables**					**DoE Matrix**		
**Formulation Factors**	**Symbol**	**Levels of Variation**			**Exp Name**	**X2.1**	**X2.2**
		**−1**	**0**	**+1**			
Poloxamer 407 ratio	X2.1	18	19.5	21	N1	18	0.2
HPMC (blend of K100:K4, 1:1) ratio	X2.2	0.2	0.5	0.8	N2	19.5	0.2
**Dependent Variables**					N3	21	0.2
					N4	18	0.5
**Responses**			**Symbols**		N5	19.5	0.5
Spreadability (mm)			Y2.1		N6	21	0.5
Viscosity at 25 °C (mPa·s)			Y2.2		N7	18	0.8
Viscosity at 37 °C (mPa·s)			Y2.3		N8	19.5	0.8
Viscosity of diluted gel with water 37 °C (mPa·s)			Y2.4		N9	21	0.8
Detachment force (mN)			Y2.5		N10	19.5	0.5
Bioadhesion force (Pa)			Y2.6		N11	19.5	0.5
*k* Peppas for metronidazole release (h^−n^)			Y2.7		N12	19.5	0.5
*n* Peppas for metronidazole release			Y2.8				
*k* Peppas for ibuprofen release (h^−n^)			Y2.9				
*n* Peppas for ibuprofen release			Y2.10				

**Table 10:** Mathematical models tested for release kinetics estimation.

Mathematical Model Name	Equation
Baker–Lonsdale	(3/2)[1 − (1 − (*Q_t_*/*Q_∞_*)^2/3^] − (*Q_t_*/*Q_∞_*) = *Kt*
Korsmeyer–Peppas	*Q_t_*/*Q_∞_* = *Kt^n^*
Hixon–Crowell	*Q*_0_^1/3^ − *Q_t_*^1/3^ = *Kt*
Higuchi	*Q_t_*/*Q_∞_* = *K t*^0.5^
First order	*Q_t_*/*Q_∞_* = *K t*
Zero order	*Q_t_* = *Q*_0_ + *Kt*

## Data Availability

The raw data supporting the conclusions of this article will be made available by the authors upon request.

## Supplementary Materials

The following supporting information can be downloaded at: https://www.mdpi.com/article/10.3390/gels12040331/s1, Figure S1: Fitting of the viscosity data of optimized formulation with the Power Law; Table S1: Experimental data from in vitro release of metronidazole from mucoadhesive thermosensitive gel based on Poloxamer 407 and HPMC K100M:HPMC K4M, 1:1; Table S2: Experimental data from in vitro release of ibuprofen from mucoadhesive thermosensitive gel based on Poloxamer 407 and HPMC K100M:HPMC K4M, 1:1; Table S3: Results obtained from fitting of metronidazole release data with kinetic equations; Table S4: Results obtained from fitting ibuprofen release data with different kinetic equations.

## Abbreviations

The following abbreviations are used in this manuscript:

AO	Alveolar Osteitis
APIs	Active Pharmaceutical Ingredients
CQAs	Critical Quality Attributes
DoE	Design of Experiments
DS	Design Space
FMEA	Failure Mode and Effects Analysis
HPMC	Hydroxypropyl Methylcellulose
IB	Ibuprofen
MTBG	Mucoadhesive Thermosensitive Buccal Gel
MZ	Metronidazole
PEO	Polyethylene oxide
PLS	Partial Least Squares
QbD	Quality by Design
QTPP	Quality Target Product Profile
RPN	Risk Priority Number
