# Supplementary material for: QbD Approach for Development of a Mucoadhesive Thermosensitive Gel for Oral Application: Risk Assessment Followed by Screening and Optimization
Source: Gels. 2026 Apr 16;12(4):331. doi: 10.3390/gels12040331 (PMC13115452; doi:10.3390/gels12040331)
Supplement: Supplementary file 1 [file gels-12-00331-s001.zip › gels-4204376-supplementary.pdf]

Figure S1. Fitting of the viscosity data of optimized formulation with the Power Law

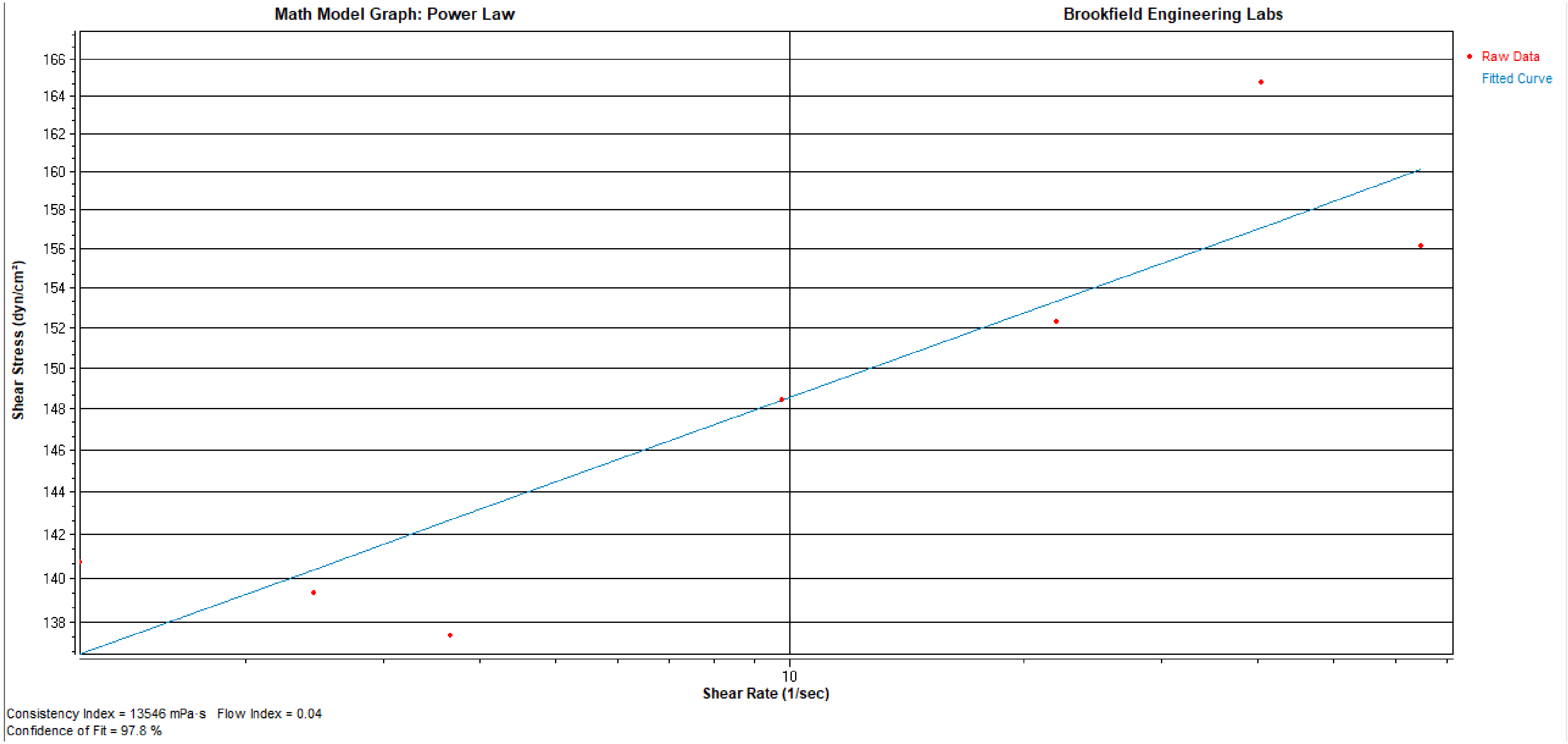

Table S1. Experimental data from in vitro release of metronidazole from mucoadhesive thermosensitive gel based on Poloxamer 407 and HPMC K100 : HPMC K4, 1:1

Time (h)	N1	N2	N3	N4	N5	N6	N7	N8	N9	N10	N11	N12
0.5	27.60	22.33	21.77	22.40	22.26	20.27	22.60	21.60	21.09	20.60	22.36	21.92
1	53.44	51.70	52.59	52.60	53.01	50.17	52.40	47.20	45.88	49.20	54.00	50.10
2	79.63	79.74	78.74	77.90	77.70	74.28	72.54	68.25	63.89	76.82	77.80	76.66

4	98.84	97.82	97.30	92.30	91.94	88.26	86.22	84.23	79.92	90.82	92.45	91.82
6	99.82	99.55	98.18	95.70	95.33	93.20	94.88	91.12	87.10	95.66	95.48	95.57
8	100.00	100.00	100.00	96.80	96.50	95.33	96.45	93.24	91.12	96.80	96.83	97.10

Table S2. Experimental data from in vitro release of ibuprofen from mucoadhesive thermosensitive gel based on Poloxamer 407 and HPMC K100 : HPMC K4, 1:1

Time (h)	N1	N2	N3	N4	N5	N6	N7	N8	N9	N10	N11	N12
0.5	9.20	8.70	4.58	5.60	4.92	4.10	4.59	5.55	3.40	5.20	6.11	4.62
1	11.26	10.32	7.13	7.78	7.83	5.80	7.88	7.24	5.22	8.10	9.21	6.83
2	19.32	15.42	13.10	16.30	15.20	12.39	16.16	15.00	8.22	15.22	16.21	15.84
4	35.50	22.60	24.10	31.00	27.80	22.66	30.62	27.70	19.33	24.20	28.11	27.65
6	51.60	34.90	35.10	43.50	41.30	32.46	43.10	40.10	26.30	42.30	43.22	41.89
8	63.80	42.65	41.70	56.90	48.20	44.21	55.55	47.20	34.00	49.20	48.97	47.66

Table S3. Results obtained from fitting of metronidazole release data with kinetic equations

	Baker and Lonsdale			Peppas				Hixon and Crowell			Higuchi		
	R	AIC	K	R	AIC	k	n	R	AIC	k	R	AIC	k
N1	0.9752	24.90	0.0733	0.9885	18.88	50.5569	0.5121	0.9992	14.86	0.2100	0.9884	16.91	51.1

N2	0.9624	26.65	0.0685	0.9813	25.89	47.9777	0.5493	0.9973	16.16	0.2020	0.9797	24.22	50.1199
N3	0.9626	26.59	0.0677	0.9806	25.99	47.8268	0.5469	0.9963	17.35	0.2006	0.9791	24.29	49.8592
N4	0.9676	25.68	0.0638	0.977	26.32	47.8290	0.5141	0.9928	19.70	0.1969	0.9768	24.35	48.4275
N5	0.9752	25.68	0.0629	0.9762	26.43	47.8681	0.511	0.9919	20.15	0.1968	0.9761	24.45	48.335
N6	0.9661	25.52	0.056	0.9753	26.28	45.2108	0.5231	0.9871	21.71	0.1776	0.9749	24.34	46.1433
N7	0.9743	24.16	0.0557	0.9772	25.70	46.4094	0.4846	0.9802	23.12	0.1786	0.977	23.73	45.7877
N8	0.9767	23.47	0.0485	0.9839	24.01	43.0576	0.5158	0.9823	22.39	0.1561	0.9837	22.05	43.6605
N9	0.9859	27.46	0.0423	0.9831	30.36	42.2493	0.4338	0.9667	31.71	0.1358	0.9791	29.41	38.8398
N10	0.9633	26.09	0.059	0.9761	26.40	45.6625	0.5376	0.992	20.05	0.1842	0.9751	24.56	47.2062
N11	0.9676	25.69	0.0647	0.976	26.50	48.2886	0.508	0.9918	20.23	0.1996	0.976	24.51	48.6316
N12	0.9671	25.66	0.0608	0.9792	25.85	46.4318	0.5302	0.9937	19.12	0.1879	0.9786	23.97	47.6871

Table S4. Results obtained from fitting ibuprofen release data with different kinetic equations

	**Baker and Lonsdale**			**Peppas**				**Hixon and Crowell**			**Higuchi**		
	**R**	**AIC**	**K**	**R**	**AIC**	**k**	**n**	**R**	**AIC**	**k**	**R**	**AIC**	**k**
N1	0.945	37.35	0.008	0.9985	17.90	11.6363	0.8198	0.9972	19.70	0.0356	0.9611	35.32	19.6714
N2	0.9678	28.82	0.0035	0.9932	21.61	9.7449	0.6996	0.9792	26.23	0.022	0.9754	27.22	13.4697
N3	0.9502	32.04	0.0033	0.9988	11.74	7.67	0.8252	0.9977	13.74	0.0216	0.9595	30.83	13.0706
N4	0.9338	37.17	0.0057	0.9996	8.74	8.7562	0.8993	0.999	11.92	0.0296	0.9483	35.73	16.9433
N5	0.9453	34.47	0.0045	0.9981	16.61	8.7372	0.8357	0.9982	14.23	0.0258	0.9567	33.10	15.1581
N6	0.9319	34.14	0.0031	0.9993	8.82	6.2194	0.9367	0.9985	11.46	0.0213	0.9425	33.16	12.8417
N7	0.9344	36.93	0.0055	0.9998	4.45	8.652	0.8963	0.9997	4.76	0.029	0.9485	35.53	16.647
N8	0.9473	33.94	0.0043	0.998	16.48	8.7	0.8265	0.9977	15.23	0.0251	0.9583	32.57	14.8606
N9	0.937	30.82	0.0019	0.998	12.17	5.1524	0.9105	0.9981	10.05	0.0163	0.9448	30.05	10.1627
N10	0.9345	35.64	0.0044	0.9948	22.64	8.0074	0.8838	0.9945	21.00	0.0257	0.9467	34.44	15.0918
N11	0.9515	33.88	0.0048	0.9969	19.58	9.6697	0.7954	0.9954	19.93	0.0267	0.9627	32.35	15.6767
N12	0.9428	34.79	0.0045	0.9963	20.58	8.6914	0.8377	0.9969	17.48	0.0257	0.9542	33.50	15.126
